# Association of Vitamin D Status with Immune Markers in a Cohort of Healthy Adults

**DOI:** 10.1016/j.tjnut.2024.12.010

**Published:** 2024-12-21

**Authors:** Niknaz Riazati, Reina Engle-Stone, Charles B Stephensen

**Affiliations:** 1 Graduate Group of Molecular, Cellular, and Integrative Physiology, University of California, Davis, Davis, CA, United States; 2 USDA Western Human Nutrition Research Center, University of California, Davis, Davis, CA, United States; 3 Department of Nutrition, University of California, Davis, Davis, CA, United States

**Keywords:** 25-hydroxy vitamin D, healthy adults, innate and adaptive immune responses, observational study, vitamin D status

## Abstract

**Background:**

Immune function is affected by vitamin D status, but the optimal serum 25-hydroxy vitamin D [25(OH)D] concentration for immune function is not known.

**Objectives:**

We hypothesized that 25(OH)D would be associated with markers of inflammation and immune activation.

**Methods:**

We identified associations between 25(OH)D and immune markers from 361 healthy adults using polynomial regression. Linear regression was used to define the slope (β) of significant linear associations, and piecewise regression identified inflection points (IPs) for curvilinear associations with *P* < 0.05. IPs with a slope difference (SD) *P* < 0.05 before and after were significant.

**Results:**

25(OH)D had linear, negative associations with interleukin (IL)-6 (β: −0.126; *P* = 0.009) and macrophage-derived chemokine (MDC) (β: −0.108; *P* = 0.04) and a linear, positive association with matrix metalloproteinase (MMP)-1 (β: 0.108; *P* = 0.04). Among the significant curvilinear associations, 2 showed negative associations below but positive associations above an IP with nearly significant SD *P* values, including percentage of effector-memory CD8 T cells (IP: 56.2 nmol/L; SD *P* = 0.067) and platelet concentration (IP: 38.9 nmol/L; SD *P* = 0.058). The opposite associations, positive below and negative above an IP, were seen for eotaxin (IP: 49.5 nmol/L; SD *P* = 0.049); interferon (IFN)-γ-induced protein-10 (IP-10) (IP: 71.8 nmol/L; SD *P* = 0.02); percentage of CD4 T cells expressing programmed cell death protein (PD)-1 (IP: 71.2 nmol/L; SD *P* = 0.01); percentage of Tregs expressing human leukocyte antigen, DR isotype (HLA-DR) (IP: 67.5 nmol/L; SD *P* < 0.0001); percentage of memory Tregs (IP: 68.8 nmol/L; SD *P* = 0.002); and percentage of memory Tregs expressing HLA-DR (IP: 68.8 nmol/L; SD *P* = 0.0008).

**Conclusions:**

These findings are consistent with low vitamin D status allowing and higher vitamin D status dampening inflammation and immune activation. IP analysis identified possible thresholds for vitamin D effects on immune function. Two of 3 IPs at ∼50 nmol/L show higher inflammation below this concentration, suggesting 50 nmol/L as a minimum target for dampening inflammation. IPs at ∼70 nmol/L identify a threshold for CD4 T-cell activity, including Treg activation and IFN-γ-driven production of the T-cell chemokine IP-10, suggesting an optimal concentration for regulating adaptive immunity.

This study was registered at clinicaltrials.gov as NCT02367287.

## Introduction

Vitamin D_3_ (cholecalciferol) is synthesized from the cholesterol precursor, 7-dehydrocholesterol in the skin by exposure to UV radiation. Vitamin D is supplied by diet in the form of vitamin D_2_ or ergocalciferol and is present in foods such as oil-rich fish and mushrooms. Vitamin D-fortified foods and beverages as well as dietary supplements contain both D_2_ and D_3_ forms [1,2]. Both vitamin D_2_ and D_3_ are metabolized through 2 hydroxylation steps to form the active metabolite, 1,25-dihydroxy vitamin D [1,25(OH)_2_D]. The first hydroxylation occurs in the liver, where vitamin D is converted to 25-hydroxy vitamin D [25(OH)D]. 25(OH)D has minimal biological activity and is the most abundant form of vitamin D in circulation [3,4]. The second hydroxylation occurs in the kidneys as well as other tissues including cells of the immune system, where 25(OH)D is converted to 1,25(OH)_2_D, or calcitriol, which binds to the vitamin D receptor (VDR) in these cells to regulate gene transcription [5].

Serum or plasma 25(OH)D concentration is used to assess vitamin D status (i.e., an index of the total body stores of vitamin D). The cutoff concentrations for evaluation of vitamin D status were defined separately by the United States Institute of Medicine (IOM) and the Endocrine Society (ES) based on calcium absorption, bone mineralization, and overall skeletal health, although there has not been a consensus on how to determine which 25(OH)D concentrations define vitamin D status in healthy and diseased populations [[6], [7], [8], [9]]. The IOM defined a concentration of 25(OH)D <30 nmol/L as deficiency, 25(OH)D ≥30 to <50 nmol/L as insufficiency, and 25(OH)D ≥50 nmol/L as sufficiency, whereas the ES previously defined a concentration of 25(OH)D ≥25 to <50 nmol/L as deficiency, ≥50 to <75 nmol/L as insufficiency, and ≥75 nmol/L as sufficiency [8,9]. However, the ES no longer endorses specific 25(OH)D concentrations to define deficiency, insufficiency, or sufficiency or specific concentrations for other specific health outcomes, such as immune function [10].

Although based on skeletal health outcome, a serum 25(OH)D concentration of ≥50 nmol/L covers the requirements of general healthy population [[11], [12], [13]], but some data suggest that higher concentrations of 25(OH)D may be required for optimal risk reduction for various endpoints [13,14]. Examples are vitamin D-deficient patients with rickets and osteomalacia or chronic conditions such as chronic kidney disease [15], as well as other potentially at-risk groups such as individuals with obesity or women with breast cancer [16] or during pregnancy and lactation [17,18].

In addition to the kidneys, various types of immune cells including lymphocytes, monocytes, macrophages, and dendritic cells (DCs) express VDR and vitamin D-metabolizing enzymes and therefore, cells of the immune system can metabolize 25(OH)D to calcitriol and can regulate gene expression via VDR [19]. Previous studies have shown that vitamin D modulates immune functions through intracrine and paracrine mechanisms [20,21]. Also, it has been shown that multiple diseases related to the immune system, such as autoimmune disorders and infectious diseases, develop because of low plasma concentrations of vitamin D [[22], [23], [24], [25]]. However, it is still controversial what concentration of 25(OH)D is optimal for the immune health-related outcomes [15,26,27]. In this study, we evaluated the relationship between serum 25(OH)D and biomarkers of innate and adaptive immunity. We hypothesized that 25(OH)D concentration would be associated with biomarkers of the innate and adaptive immune responses in healthy individuals. Regression methods were used to identify associations, linear or curvilinear, between serum 25(OH)D and these markers. Furthermore, we sought to identify the transition points, known as the breakpoints, in the curvilinear association of 25(OH)D with immune markers that might be suggestive of the inflection points (IPs) that would define the 25(OH)D concentrations related to normal immune function.

## Methods

### Study participants

25(OH)D concentrations were measured from 361 clinically healthy adults who participated in a nutritional phenotyping study at the USDA Western Human Nutrition Research Center (WHNRC). Participants were women and men stratified into the 3 age categories (18–34, 35–49, and 50–65 y), 3 BMI categories (18.5–24, 25–29, and 30–44 kg/m^2^), and positive/negative cytomegalovirus (CMV) infection status. The study was registered at clinicaltrials.gov as NCT02367287 [28]. The recruitment strategy, the study design and the methodology of data collection were previously described [28]. In this cross-sectional analysis, immune function markers and 25(OH)D concentration were measured using fasting blood samples. Ethical approval for this study was received from the Institutional Review Board of the University of California, Davis, as previously described [28,29].

### Biochemical methods

#### 25(OH)D

The total concentration of 25(OH)D was measured in serum from 361 participants using the Elecsys 2010 competitive binding assay run on the Cobas E-411 autoanalyzer (Roche Diagnostics GmbH) designed to quantify both 25(OH)D_2_ and 25(OH)D_3_. Serum was prepared from fasting blood collected in the morning after an overnight fast. Samples had previously been thawed once for another assay, were maintained on ice, and immediately refrozen at −80°C. The detection range of 25(OH)D was 12.5–150 nmol/L. Samples above this range were diluted 2-fold and tested again. At least 10% of samples were analyzed in duplicate on each run of the autoanalyzer. Mean coefficients of variation for each run were <4%. External validity of the assay was confirmed by running standards supplied by the manufacturer and standard reference material provided by the United States National Institute of Standards and Technology [26]. The final concentration was indicated by nmol/L.

#### Function markers of immune activity

Seventy-nine immune variables were measured from blood samples of 361 healthy fasting adults, as described previously [30]. Blood was drawn at WHNRC in the morning after a 12-h overnight fast after consumption of a standard meal the prior evening. Immune markers were measured from plasma, cultures of peripheral blood mononuclear cells (PBMCs), and whole blood.

#### Immune markers measured in fasting plasma

The concentrations of soluble immune markers in plasma, including neopterin, myeloperoxidase, and soluble CD14, were measured by ELISA. The concentrations of 16 plasma proteins were measured using the MSD U-plex Platform: C-reactive protein, serum amyloid A, intracellular cell adhesion molecule-1, vascular cell adhesion molecule-1, eotaxin, interferon (IFN)-γ-induced protein-10 (IP-10), monocyte chemoattractant protein-1, macrophage-derived chemokine (MDC), TNF-α, IL-1β, IL-6, IL-8, IL-10, matrix metalloproteinase (MMP)-1, MMP-3, and MMP-9.

#### Cytokine concentrations measured from PBMC cultures

Soluble immune markers were also measured in supernatants of PBMCs using MSD assay. The concentrations of cytokines, including TNF-α, IL-1β, IL-6, and IL-10, were measured from PBMCs cultured with lipopolysaccharide (LPS) from *E. coli* 0111: B4. Moreover, PBMC cultures were stimulated with anti-CD3 plus anti-CD28 monoclonal antibodies for measuring T-cell cytokines including IFN-γ, IL-10, IL-13, and IL-17A concentrations.

#### Cellular markers measured in fasting peripheral blood and a complete blood count

Cellular markers were measured using flow cytometry of peripheral blood. Leukocytes in isolated PBMCs and whole blood were analyzed using 4 flow cytometry panels: Panel A, naïve and central/effector-memory T cells recognized by activation markers CD38 and human leukocyte antigen, DR isotype (HLA-DR) and CD4 T cells by the marker of T-cell exhaustion, programmed cell death protein-1 (PD-1); Panel B, helper T-cell types Th1, Th2, and Th17, natural killer (NK) cells, NK T cells, and B cells; Panel C, total and memory Treg cells recognized by activation markers CD38 and HLA-DR; and Panel D, total T cells plus monocytes (classic, intermediate, and alternative), neutrophils, and eosinophils recognized by activation marker CD11b. complete blood count with differential was used to measure platelets, neutrophils, lymphocytes, monocytes, eosinophils and basophils.

The immune markers were defined using the following categories: effector/memory T cells and activation levels (*n* = 24), other lymphocytes including helper T cells, NK cells, NK T cells, and B cells (*n* = 11), PBMC cytokines (*n* = 8), complete blood count (*n* = 6), innate cell activation (*n* = 11), and plasma markers (*n* = 19), as summarized in Supplemental Table 1 and described previously [30]. To achieve a normal distribution, all immune variables were normalized using rank-based normal transformation.

#### CMV antibody test

Multiple studies have shown that reactivation of CMV infection is associated with development of activated CD4 and CD8 T-cell profiles [31,32]. For this reason, we controlled our preliminary regression analysis of the association of vitamin D status with markers of immune activity for potential differences of the immune activity between CMV seropositive and seronegative individuals by measuring CMV-specific IgG status, as described previously [33].

### Statistical methods

To identify associations between serum 25(OH)D concentrations and immune markers, we conducted polynomial regression analyses with quadratic and cubic terms of log-25(OH)D. When curvilinear associations were statistically significant, a piecewise regression model was used to identify transitions between zero and nonzero slopes.

We inspected the scatter plots of the regression models for outliers, removed the outliers, and re-ran piecewise regression analysis to identify the associations that survived the outlier removal. The analyses of this study were intended to be broadly descriptive of the association of vitamin D status with immune function. Thus, a standard *P* value <0.05 was used without correction for multiple comparison among the immune markers.

All statistical analyses were conducted in SAS 9.4 (SAS Institute). Association of 25(OH)D with immune variables were plotted using SAS (FIGURE 1, FIGURE 2, FIGURE 3, FIGURE 4). GraphPad Prism v9.5.0 was used for creating Figure 5 summarizing the IPs in the curvilinear associations between 25(OH)D and immune markers.

**FIGURE 1:**
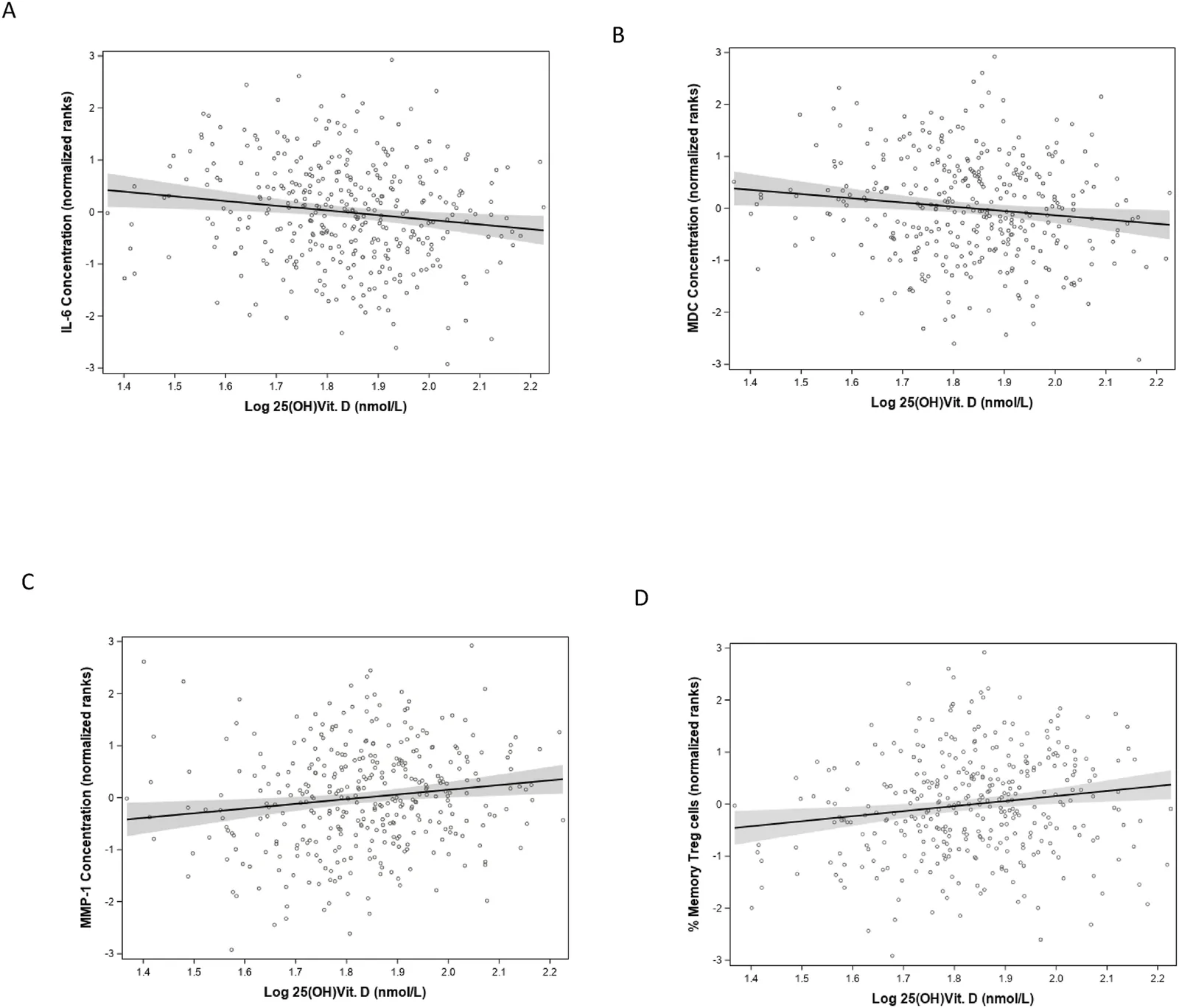
Linear associations between 25(OH)D and plasma concentrations of IL-6, MDC, and MMP-1 and percentage of memory Tregs. 25(OH)D was negatively associated with (A) IL-6 (β: −0.126; *P* = 0.009) and (B) MDC (β: −0.108; *P* = 0.04) and positively associated with (C) MMP-1 (β: 0.108; *P* = 0.04) and (D) memory Treg percentage (β: 0.103; *P* = 0.02). 25(OH)D, 25-hydroxy vitamin D; MDC, macrophage-derived chemokine; MMP, matrix metalloproteinase.

**FIGURE 2:**
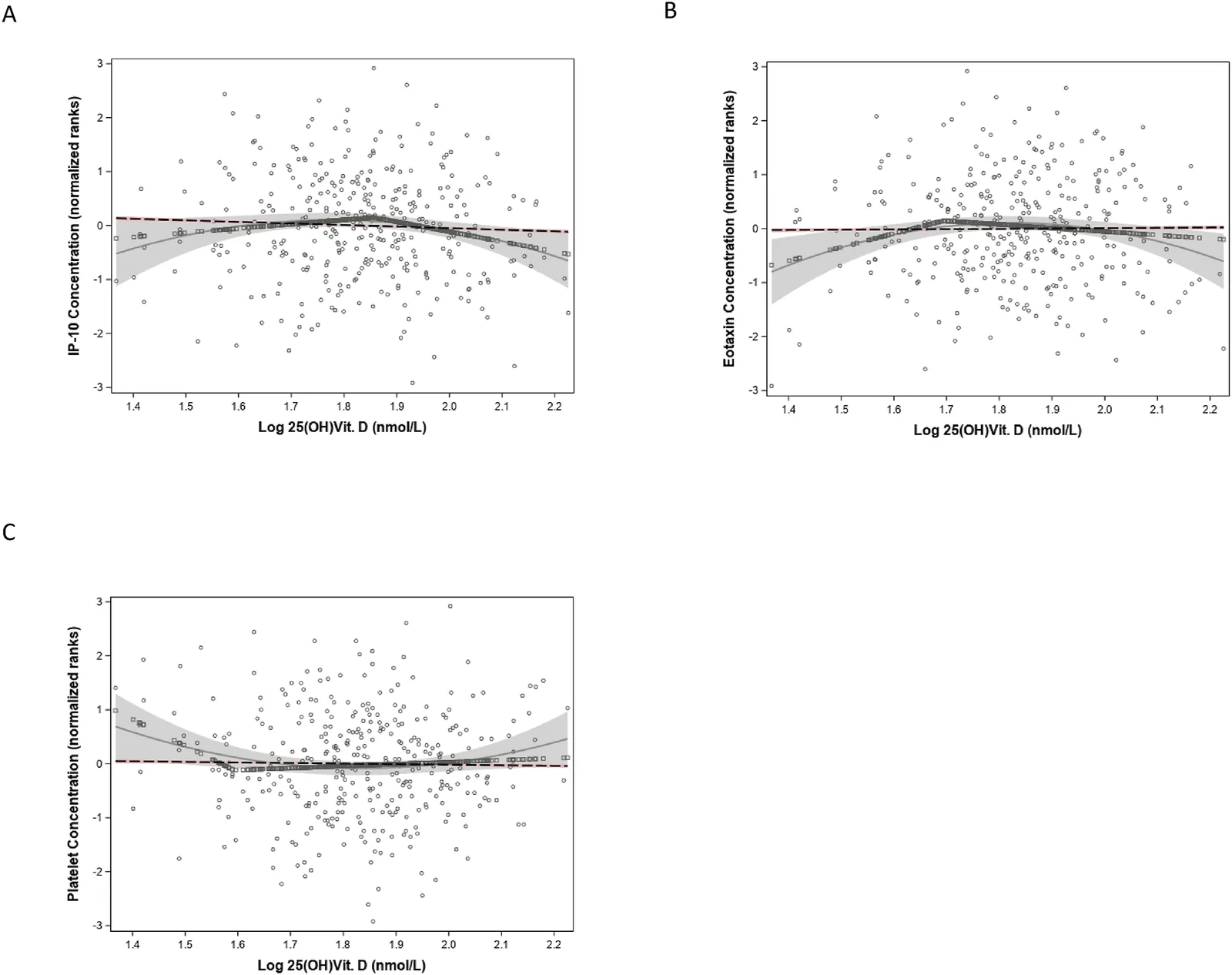
Curvilinear associations between 25(OH)D and markers of innate immune response including IP-10, eotaxin, and platelets identified by second-order polynomials. The grey line represents the linear regression model across the observed range of the immune markers fitted by the quadratic function. The shaded area represents 95% CIs for the polynomial regression model. The black line is the regression line, and the squares are piecewise regression-predicted values of the immune markers. (A) 25(OH)D >71.8 nmol/L [log-25(OH)D = 1.86] was negatively associated with plasma concentration of IP-10. (B) 25(OH)D <49.5 nmol/L [log-25(OH)D = 1.69] was positively associated with eotaxin concentration. (C) 25(OH)D <38.9 nmol/L [log-25(OH)D = 1.59] was negatively associated with blood concentration of platelets. 25(OH)D, 25-hydroxy vitamin D; CI, confidence interval; IP-10, interferon γ-induced protein 10.

**FIGURE 3:**
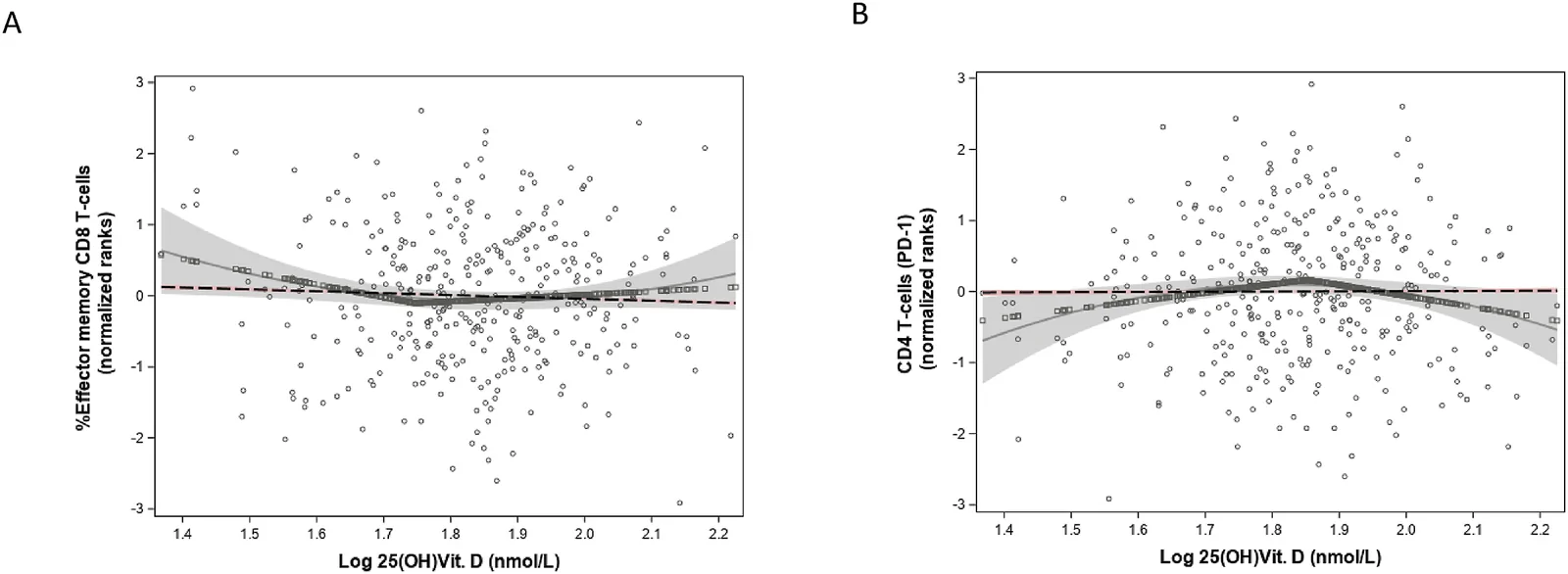
Curvilinear associations between log-25(OH)D and markers of the adaptive immune response identified by second-order polynomial regressions. The grey line represents the linear regression model across the observed range of the immune markers fitted by the quadratic function. The shaded area represents 95% CIs for the polynomial regression model. The black line is the regression line, and the squares are piecewise regression-predicted values of the immune markers. (A) 25(OH)D <56.2 nmol/L [log-25(OH)D <1.75] was negatively associated with the percentage of effector-memory CD8 T cells. (B) 25(OH)D >71.2 nmol/L [log-25(OH)D >1.85] was negatively associated with the percentage of CD4 PD-1^+^ T cells. 25(OH)D, 25-hydroxy vitamin D; CI, confidence interval; PD-1, programmed cell death protein 1.

**FIGURE 4:**
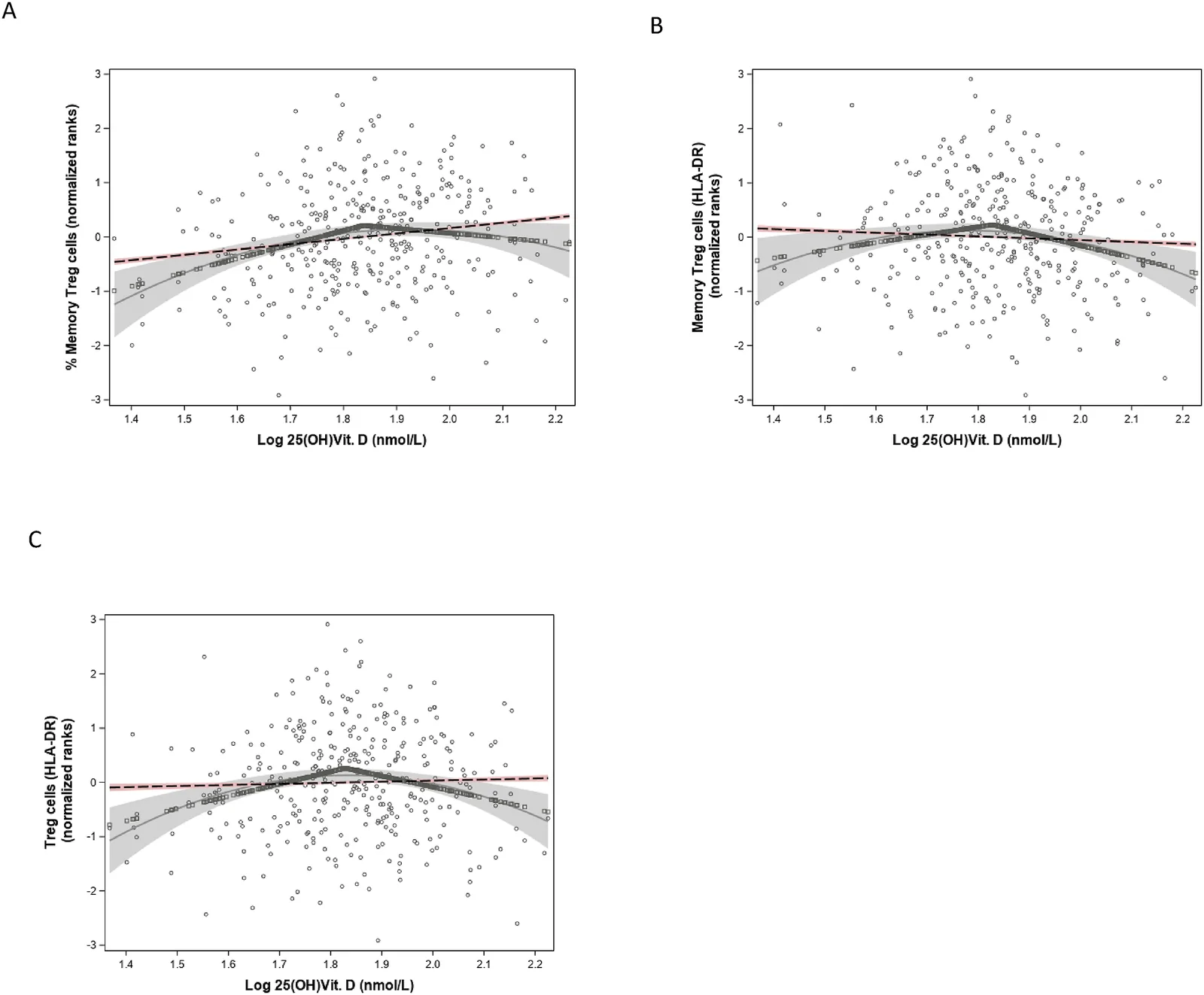
Curvilinear associations between 25(OH)D and markers of regulatory immune function identified by second-degree polynomial regressions. The grey line represents the linear regression model across the observed range of the immune markers fitted by the quadratic function. The shaded area represents 95% CIs for the polynomial regression model. The black line is the regression line, and the squares are piecewise regression-predicted values of the immune markers. (A) 25(OH)D >68.8 nmol/L [log-25(OH)D >1.84] was negatively associated with the percentage of memory Tregs. (B) 25(OH)D >67.4 nmol/L [log-25(OH)D >1.83] was negatively associated with the percentage of HLA-DR^+^ memory Tregs. (C) 25(OH)D >67.5 nmol/L [log-25(OH)D >1.83] was negatively associated with the percentage of HLA-DR^+^ Tregs. 25(OH)D, 25-hydroxy vitamin D; CI, confidence interval; HLA-DR, human leukocyte antigen, DR isotype.

**FIGURE 5:**
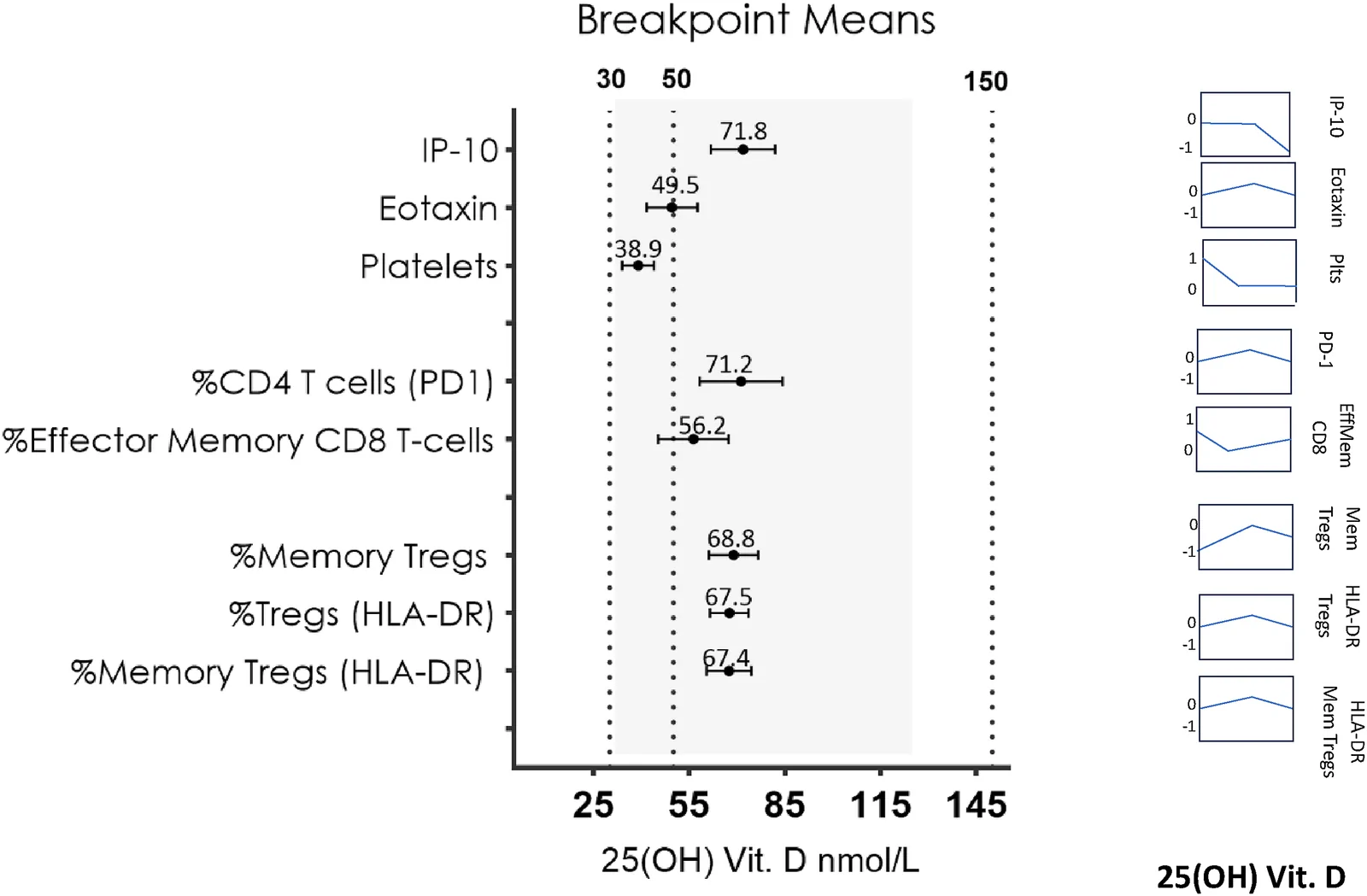
The mean ± SE for the breakpoint concentrations of 25(OH)D. Shown on the top of the graph are the cutoff concentrations of 25(OH)D that determine deficient, insufficient, sufficient, and excess concentrations of 25(OH)D as defined by the Institute of Medicine (<30, 30–50, ≥50, and ≥150 nmol/L, respectively). Breakpoints were identified between 36.6 and 120.6 nmol/L, which correspond to the 5th and 95th percentiles of 25(OH)D concentrations in our dataset, identified by shading. The direction indicator graphs on the right side show the direction of the associations below and above the breakpoint concentrations shown on the left side. 25(OH)D, 25-hydroxy vitamin D; EffMem CD8, effector-memory CD8 T cell; HLA-DR, human leukocyte antigen, DR isotype; Mem Treg, memory Treg cell; PD1, programmed cell death protein 1; Plts, platelets.

#### Covariates

The regression models were adjusted for covariates using categorical variables for age, BMI, sex, and CMV infection status. In addition, to determine whether the regression models should also be adjusted for the interaction terms of the covariates age, BMI, and sex, we determined whether there was a significant relationship between the interaction terms of the covariates and 25(OH)D. To do this, an analysis of variance was conducted using 2- and 3-way interaction terms of age and BMI categories and sex (independent variables) and rank-normalized 25(OH)D concentrations (dependent variable). None of the 2-way and 3-way interaction terms of the covariates were significantly associated with 25(OH)D (data not shown); thus, we did not use the interaction terms of the covariates in our analyses.

#### Polynomial regression analysis

25(OH)D was tested for normal distribution using the Shapiro-Wilk goodness-of-fit test. Log_10_-transformed values improved normal distribution (*W* = 0.99; *P* = 0.13). To examine associations between 25(OH)D concentrations and immune markers, a polynomial regression model was fitted to the data using PROC GLM in SAS. This preliminary analysis was performed with linear, quadratic, and cubic functions of log-normalized 25(OH)D (the predictor variable) to identify immune markers (the response variables) that were associated with 25(OH)D concentrations. Polynomial associations with *P* values of <0.05 (probability of type I sum of squares) in the model were considered significant. When the polynomial models identified a significant linear association, a generalized linear regression model was used to obtain the β coefficient. Before the linear regression analysis, the immune variables were standardized by rank-normalization, and the log-25(OH)D values were standardized using PROC Standard in SAS. The regression coefficients are reported by standardized β. When both linear and curvilinear associations were significant, a polynomial contrast was conducted to assess whether adding the second- and third-degree polynomials would improve the model fit significantly. Second- and third-degree polynomial coefficients that were different from zero with a *P* value of <0.05 indicated that a curvilinear model would be a better fit to our dataset.

#### Piecewise linear regression

The scatter plots of the second- and third-degree polynomial regressions that were identified as significant were visually inspected to obtain initial estimates of the IPs known as the breakpoints, in the association of 25(OH)D with immune variables. To determine the location of a breakpoint, we used PROC NLIN to fit a segmented regression model for the curvilinear association [34]. The model consists of 2 straight lines that join smoothly at an unknown breakpoint at which the concentration of 25(OH)D marks the transition of one linear regression line to the next one. To determine whether the individual regression slopes before and after the breakpoints were significantly different from each other, a slope difference (SD) test was conducted. The regression slopes before and after the breakpoints were compared using the ESTIMATE statement in the piecewise regression model. The significance of the SD was determined by *P* < 0.05.

Due to the small number of study participants with 25(OH)D deficiency (<30 nmol/L) and excess (≥150 nmol/L), the breakpoints identified at these concentrations are not shown in this article, as further assessment is required to confirm these results. To ensure that the breakpoints discussed in this study were not affected by this limitation, we set the minimum of 36.6 nmol/L and the maximum of 120.6 nmol/L as the reliable lowest and highest breakpoint values, which correspond to the 5th and 95th percentiles of 25(OH)D concentrations in our dataset.

## Results

The mean ± SEM total concentration of 25(OH)D in our study participants was 72.6 ± 1.36 nmol/L. Table 1 shows the participant characteristics stratified by sex, the 3 age and 3 BMI groups, and CMV infection status. Table 2 shows the distribution of our study participants by sex, the 3 age and BMI groups, and CMV infection status across the categories of 25(OH)D concentrations defined as deficient, insufficient, sufficient, and excess by the IOM.

**TABLE 1:** Summary of participant characteristics grouped by sex, age, BMI, and CMV infection status.

Categories	*n*	Percentage
All participants (*N*)	361	100
Sex		
Females	190	52.6
Males	171	47.4
Age (y)		
18–34	129	35.7
35–49	118	32.7
50–66	114	31.6
BMI (kg/m^2^)		
18–24	139	38.5
25–29	131	36.3
30–44	91	25.2
CMV IgG		
Positive	178	49.3
Negative	182	50.4

Abbreviations: BMI: body mass index; CMV: cytomegalovirus.

**TABLE 2:** Participant categories of age, BMI, sex, and CMV-specific IgG status characterized by the IOM vitamin D status.

25(OH)D (nmol/L)	Age (y)			BMI (kg/m^2^)			Sex		CMV		All (%)
	18–34	35–49	50–66	18–24	25–29	30–44	Females	Males	IgG^-^	IgG^+^	
Deficiency (<30)	3	3	0	3	1	2	3	3	4	2	1.7
Insufficiency (30–50)	30	16	11	17	22	18	25	32	28	29	15.8
Sufficiency (50–150)	96	96	103	118	107	70	160	135	149	145	81.7
Excess (≥150)	0	3	0	1	1	1	2	1	1	2	0.83

Abbreviations: 25(OH)D, 25-hydroxy vitamin D; BMI, body mass index; CMV, cytomegalovirus; IOM, Institute of Medicine.

Our regression analysis identified 4 linear associations between 25(OH)D and the concentrations of immune markers IL-6, MDC, and MMP-1 and percentage of memory Tregs. The curvilinear associations that survived the slope significance test were identified as significant. They include the associations between 25(OH)D and 8 immune markers including IP-10, eotaxin, and platelet concentrations and percentages of CD4 PD-1^+^ T cells, effector-memory CD8 T cells, total memory Tregs, HLA-DR^+^ Tregs, and HLA-DR^+^ memory Tregs. 25(OH)D was either not significantly associated with the other immune markers tested or the associations identified by the preliminary polynomial regression, including those with percentages of CD4 T cells, CD8 T cells, Th2 cells, and Th17 cells and MPO concentration did not survive the slope significance test.

### Association of 25(OH)D with immune markers identified by polynomial regression model

The association of 25(OH)D with IL-6 and MMP-1 concentrations and memory Treg percentage were identified as significant by both linear and curvilinear models. The polynomial contrast indicated that second- and third-order polynomials did not improve the model fit for IL-6 and MMP-1 (contrast *P* = 0.05), but they improved the model fit for memory Treg percentage (contrast *P* = 0.03). Log-25(OH)D was negatively associated with IL-6 (β: −0.126; *P* = 0.009) (Figure 1A) and MDC (β: −0.108; *P* = 0.04) (Figure 1B). In addition, log-25(OH)D was positively associated with MMP-1 concentration (β: 0.108; *P* = 0.04) (Figure 1C) and percentage of memory Tregs (β: 0.103; *P* = 0.02) (Figure 1D). No other linear association was found between 25(OH)D and the immune markers examined in this study.

The curvilinear associations identified by second-order polynomials include the association of log-25(OH)D with markers of innate immune response including plasma concentrations of IP-10 (*P* = 0.01) (Figure 2A), eotaxin (*P* = 0.01) (Figure 2B), and blood concentration of platelets (*P* = 0.049) (Figure 2C); markers of the adaptive immune response including percentage of effector-memory CD8 T cells (*P* = 0.01) (Figure 3A) and percentage of CD4 PD-1^+^ T cells (*P* = 0.04) (Figure 3B); and markers of immune regulation including percentage of memory Tregs (p=0.01) (Figure 4A), percentage of HLA-DR^+^ memory Tregs (*P* = 0.01) (Figure 4B), and percentage of HLA-DR^+^ Treg cells (*P* = 0.002) (Figure 4C).

### Breakpoints in the curvilinear associations between 25(OH)D and markers of the innate immune response

Piecewise regression analysis showed a breakpoint in the association of 25(OH)D with IP-10 with a mean at the log-25(OH)D of 1.86 (95% confidence interval [CI]: 1.73, 1.99), which corresponds to 71.8 nmol/L. 25(OH)D was associated with a lower IP-10 concentration above but not below 71.8 nmol/L 25(OH)D (Figure 2A). Further examination of the breakpoint concentration by the regression slope test indicated that the slopes of the regression lines before and after the 25(OH)D breakpoint were significantly (*P* < 0.05) different from each other, with a *P* value of 0.02.

In addition, 25(OH)D was associated with a lower concentration of eotaxin below but not above the log-25(OH)D breakpoint of 1.69 (95% CI: 1.55, 1.84,) corresponding to 49.5 nmol/L. The association plateaued above the breakpoint (Figure 2B). Further, the SD *P* value of 0.049 indicated a significant difference between the regression slopes before and after the identified breakpoint.

The blood concentration of platelets was higher below but not above the log-25(OH)D breakpoint of 1.59 (95% CI: 1.48, 1.70), corresponding to 38.9 nmol/L. Above this concentration, the association between 25(OH)D and platelets plateaued (Figure 2C). The SD *P* value of 0.058 indicated that the slopes of the regression lines before and after the breakpoints were almost significantly different.

### Breakpoints in the associations between 25(OH)D and markers of the adaptive immune response

Using piecewise regression analysis, we found that the association of log-25(OH)D with the percentage of effector-memory CD8 T cells was higher below but not above the log-25(OH)D of 1.75 (95% CI: 1.58, 1.91), corresponding to 56.2 nmol/L (Figure 3A). The association of 25(OH)D with the percentage of CD4 PD-1^+^ T cells shifted from positive to negative at the log-25(OH)D breakpoint of 1.85 (95% CI: 1.73, 1.98), which corresponds to 71.2 nmol/L (Figure 3B). Furthermore, *P* values from the regression slope test indicated that the regression slope was significantly different before and after the 25(OH)D breakpoint associated with the percentage of CD4 PD-1^+^ T cells (SD *P* = 0.01) and almost significantly different before and after the 25(OH)D breakpoint associated with the percentage of effector-memory CD8 T cells (SD *P* = 0.067).

### Breakpoints in the associations between 25(OH)D and markers of regulatory immune response

We identified a breakpoint in the association of log-25(OH)D with the percentage of memory Tregs at the log-25(OH)D of 1.84 (95% CI: 1.74, 1.93), which corresponds to 68.8 nmol/L (Figure 4A). As Figure 4A shows, there was a positive association between the percentage of memory Tregs and 25(OH)D ≤68.8 nmol/L. However, the percentage of memory Tregs fell above this threshold.

The association of 25(OH)D with percentages of HLA-DR^+^ Tregs and HLA-DR^+^ memory Tregs had a similar shift of trend pattern. There was a positive association between 25(OH)D and percentage of HLA-DR^+^ memory Tregs up to the log-25(OH)D breakpoint of 1.83 (95% CI: 1.74, 1.92), which corresponds to 67.4 nmol/L. 25(OH)D was associated with a lower percentage of HLA-DR^+^ memory Tregs above this breakpoint (Figure 4B). Similarly, there was a positive association between 25(OH)D and HLA-DR^+^ Tregs up to the log-25(OH)D breakpoint of 1.83 (95% CI: 1.75, 1.91), which corresponds to 67.5 nmol/L. 25(OH)D above this breakpoint was associated with a lower percentage of HLA-DR^+^ Tregs (Figure 4C). Furthermore, the regression slope test indicated that the slopes were significantly different between the regression lines before and after the breakpoints. The SD *P* values in the association of 25(OH)D with the percentage of total memory Tregs was 0.002, with the percentage of HLA-DR^+^ memory Tregs was 0.0008, and with the percentage of HLA-DR^+^ Tregs was <0.0001.

Figure 5 shows the 8 breakpoints ± standard error values for curvilinear associations between 25(OH)D and the immune markers identified in this analysis. These associations remained significant after conducting sensitivity analyses including outlier removal, polynomial contrast identifying the model with a better fit between the linear and curvilinear regressions, and the regression slope test. Three of the immune variables had CIs that included (plasma eotaxin concentration and percentage of effector-memory CD8 T cells) or fell below (blood platelet concentration) the IOM 50 nmol/L adequacy cutoff, suggesting that this adequacy cutoff based on calcium biology may also approximate a threshold for these immune variables. The remaining 5 breakpoints (including plasma IP-10 concentration and percentages of CD4 PD1^+^ T cells, memory Tregs, HLA-DR^+^ memory Tregs, and HLA-DR^+^ total Tregs) were higher, with means ranging from 67 to 72 nmol/L, suggesting higher thresholds for these immune variables.

## Discussion

### Linear associations between 25(OH)D and immune markers

The negative association between 25(OH)D and IL-6 found by this study is consistent with results from studies of various pathological conditions demonstrating low concentrations of 25(OH)D were associated with high plasma concentrations of IL-6 [[35], [36], [37], [38]]. Moreover, vitamin D supplementation was shown to increase 25(OH)D and decrease IL-6 concentration in patients with multiple sclerosis [39], in children with sickle cell anemia [40], and in overweight and obese individuals [41,42]. Furthermore, vitamin D administration in a mouse model of diabetes with a high concentration of IL-6 mRNA restored 25(OH)D and significantly decreased IL-6 mRNA in the mouse liver [43]. The negative association of 25(OH)D with IL-6 in our cohort of healthy adults suggests an association with lower immune activity and inflammation. As previously reported, the presence of a high-affinity receptor for vitamin D in monocytes [44] and vitamin D suppression of IL-6 production by monocytes suggest that downregulation of IL-6 might be a receptor-mediated phenomenon [45,46].

MDC/CCL22 is expressed by DCs and macrophages and binds to and activates CCR4, the chemokine receptor on Th2 cells that is supportive of humoral responses and on Tregs. MDC is modulated by environmental signals such as cytokines and microbial products [47,48]. The negative association of 25(OH)D with MDC in this study suggests that MDC production might be downregulated by 25(OH)D. This hypothesis is supported by a study showing that vitamin D treatment of the LPS-induced DCs blocked MDC production [49]. Vitamin D was shown to modulate MDC production by suppression of DC maturation, as well as by upregulation of IL-10 by DCs and induction of a cytokine profile that favors development of IL-10-producing Tregs [[50], [51], [52]].

MMP-1 or interstitial collagenase-1 is a proteolytic enzyme from a family of proteinases produced by various cell types including epithelial cells, fibroblasts, and immune cells such as macrophages and neutrophils. MMP-1 participates in degradation of the proteins of the extracellular matrix during immune cell movement, tissue repair, and wound healing [53]. Consistent with our result, a previous in vitro study of rat chondrocytes [54] and an observational study of cord blood plasma of healthy human newborns [55] reported a positive relationship between 25(OH)D and MMP. However, vitamin D was shown to downregulate the secretion of MMPs by PBMCs [56] and various other cells in ex vivo and in vitro settings [[57], [58], [59]] as well as an observational study [60]. Vitamin D regulation of MMPs was suggested to be mediated through VDR [54,58,59,61] and induction of IL-10 and prostaglandin E2 [56]. The mixed observations regarding the relationship between 25(OH)D and MMPs could be caused by differences in experimental design, cell types, and sources of stimulation. Previous studies that found the effect of vitamin D treatment on MMP secretion varies between monocytes and PBMCs [56] and between chondrocytes of the growth plate and resting zone [54] support this idea. However, our finding needs further assessment by experimental and interventional studies, as no data exists concerning the relationship between MMP-1 and vitamin D.

### Curvilinear association between 25(OH)D and immune markers

#### Association of 25(OH)D with markers of the innate immune response

IP-10 is a leukocyte chemokine induced by IFN-γ and drives Th1-mediated inflammation [62]. The negative association of 25(OH)D >71.8 nmol/L with IP-10 is consistent with the association of vitamin D with low IP-10 concentration found by previous studies of healthy and diseased individuals. For example, a study showed that vitamin D intake by a population of healthy women increased 25(OH)D and decreased IP-10 concentrations [63]. Clinical trials showed that a sufficient concentration of 25(OH)D in patients with chronic hepatitis C and pulmonary tuberculosis was associated with a significant decrease in plasma IP-10 [64,65]. Furthermore, treatment of THP-1 cells and primary monocytes with vitamin D was shown to suppress LPS-induced IP-10 production by these cells [66,67]. IP-10 expression was shown to be regulated by VDR activation [[68], [69], [70]]. Silencing *VDR* expression in infected airway epithelial cells treated with vitamin D abolished the effect of vitamin D on IP-10 expression [69]. Vitamin D treatment of *Vdr*-deficient macrophages induced by IFN-γ did not suppress the expression of IP-10 when compared to wild-type macrophages, suggesting *IP-10* may be a VDR target gene [70]. VDR-mediated suppression of IP-10 was shown to be induced by IFN-γ as well as TLR signaling, increasing the expression of vitamin D-activating enzyme Cyp27B1 through modulation of the JAK–STAT, p38 MAPK, and NF-κB pathways [69,71].

Eotaxin/CCL11 is a chemokine marker of the type 2 immune response likely elevated during inflammation when eosinophils are recruited to inflamed tissue in a parasitic infection or an allergic disease [72]. Previous studies have shown that vitamin D treatment inhibits the expression of eotaxin mRNA in mouse adipose tissue [73,74] and lowers eotaxin plasma concentration in healthy women [63]. Eotaxin was shown to be regulated through the VDR-mediated inhibition of the NF-κB signaling pathway [74]. The transition of the 25(OH)D–eotaxin association from positive to plateau at 49.5 nmol/L 25(OH)D suggests that eotaxin might be regulated at 25(OH)D higher than this threshold concentration. This result has not been reported previously and needs further assessment by experimental and intervention studies.

The higher platelet concentration associated with 25(OH)D below but not above 38.9 nmol/L is supported by cohort studies of clinically healthy adults [75] as well as overweight and obese adults with 25(OH)D <50 nmol/L [76], showing a negative association between 25(OH)D and platelet concentration in the blood. The higher platelet concentration in our study participants with low concentrations of 25(OH)D is consistent with a higher risk of inflammation with lower vitamin D status. This inference is supported by studies showing that blood platelet concentrations are higher in inflammatory conditions [77,78]. Higher platelet concentrations may result from higher concentrations of inflammatory cytokines such as IL-6 and TNF-α in those with low vitamin D status, as these cytokines contribute to platelet activation and increased release of the activated platelets from bone marrow [79,80]. In addition, higher vitamin D status can reduce platelet intracellular oxidative stress and may thus reduce platelet activation and release from bone marrow, as has been demonstrated in adults with vitamin D deficiency [81].

#### Association of 25(OH)D with markers of adaptive immune response

Vitamin D modulates T-cell responses through monocytes and DCs, which have essential enzymes to produce calcitriol, acting upon adjacent T cells. Circulating levels of calcitriol synthesized by the kidneys and the intracrine synthesis of calcitriol by T cells also activate the VDR signal directly, enhancing the expression of IL-10 mRNA in CD4 T cells [[82], [83], [84]].

The association of 25(OH)D above but not below 71.2 nmol/L with a lower percentage of CD4 PD-1^+^ T cells found by our study is consistent with reduction of PD-1 expression in vitamin D-treated CD4 T cells reported previously [85]. However, a study of patients with Crohn’s disease showing that vitamin D supplementation increased the expression of PD-1 is in contrast with our finding [85]. PD-1 is a marker of T-cell exhaustion that is expressed at high levels following a chronic infection [86]. The reason for the mixed observation could be that vitamin D-mediated regulation of CD4 T cells might be different between healthy individuals with balanced immune activity and patients with increased T-cell activity, as was previously indicated [85].

The association of 25(OH)D below but not above 56.2 nmol/L with a higher percentage of effector-memory CD8 T cells indicates an association with proliferation of CD8 T cells, possibly in response to T-cell signaling in peripheral tissues. Consistent with our finding, a study of adults aged >80 y with low concentrations of vitamin D reported a negative correlation between 25(OH)D and effector-memory CD8 T cells [87]. Our finding suggests that low concentrations of vitamin D might be a predictor of augmented T-cell activation and inflammation.

#### Association of 25(OH)D with markers of regulatory immune response

Vitamin D-mediated activation of VDR polarizes CD4 helper T cells toward a more regulatory phenotype [83,84]. Multiple studies have shown that the vitamin D metabolite calcitriol supports Treg development and have demonstrated an association between vitamin D status and higher numbers and percentages of Tregs [20,23,[88], [89], [90], [91], [92], [93], [94]]. Some of our analyses support these earlier observations. An example is the positive association between 25(OH)D and percentage of memory Tregs. However, above the breakpoint of 68.8 nmol/L, this association is no longer positive, suggesting a threshold for development of memory Tregs, which occurs with appropriate post-thymic antigenic stimulation, may be reached at ∼68.8 nmol/L. Similarly, a study showed that 25(OH)D ≤115 nmol/L in healthy women was correlated with an increased Treg percentage, but further increase ≤145 nmol/L was correlated with a decreased Treg percentage [23]. In addition, our study found a positive association between 25(OH)D and HLA-DR^+^ Tregs and HLA-DR^+^ memory Tregs below but not above 67.5 nmol/L 25(OH)D. These 2 observations, a positive slope for HLA-DR^+^ memory Treg cells that peaks near 67 nmol/L and a positive slope for total memory Tregs that peaks near 68 nmol/L, are consistent with the idea that vitamin D supports Treg development following peripheral antigenic stimulation. Furthermore, vitamin D was shown to depress the activity of antigen-presenting cells [50,[82], [83], [84],^95^]. The lower percentage of HLA-DR^+^ memory Tregs >67 nmol/L 25(OH)D might be the result of the suppression of antigen-presenting cells by 25(OH)D greater than the suggested cutoff, which might also be the reason for the lower percentage of total memory Tregs observed above this cutoff [42,[78], [79], [80],^91^].

### Strengths and limitations

It is worth noting that we cannot draw conclusions about cause-and-effect relationships from the results of this cross-sectional study. The reported associations alone do not provide evidence about functional changes of the immune system that might be related to vitamin D status. Moreover, due to the small number of individuals with vitamin D deficiency [25(OH)D <30 nmol/L], we were not able to explore the association of frank deficiency with immune markers. Another caveat is that the breakpoints with a wider variation shown by the CIs in Figure 5 might have greater interindividual variability, which might reflect the influence of multiple determinants on immune activity in addition to vitamin D status. Therefore, the 25(OH)D breakpoint means with overlapping CIs might represent the same range of 25(OH)D concentration that might be related to multiple immune markers.

One strength of the study is that we used a relatively large sample size with a large panel of immune markers and a study population of clinically healthy adults without an active infection or a chronic disease, which might affect our study endpoint. Another strength is that we adjusted the polynomial regression models for sex, age, BMI, and CMV infection status, which might affect vitamin D status and/or immune function. Setting a detection limit within the 5th to 95th percentile of our dataset for the breakpoint identification, due to the small sample size beyond these percentiles, also strengthens the reliability of our results. Another strength of the study is the sensitivity analyses that we conducted to examine the curvilinear associations. These tests include regression analyses without the outliers, polynomial contrast analyses to determine the model between the linear and curvilinear regressions with a better fit to our dataset, and examination of the breakpoints by the regression slope test.

### Conclusions

The results presented here identify potential target values (i.e., IPs) for characterizing adequate concentrations of vitamin D for maintenance of various aspects of immune function. The negative linear associations of 25(OH)D with plasma IL-6 and MDC are consistent with higher vitamin D status dampening inflammation and immune activation across the full range in which we could identify IPs (37–121 nmol/L). Figure 5 shows IPs identified within this range. Three of the immune variables in Figure 5 had CIs for IPs that included or fell below the IOM-defined vitamin D adequacy cutoff [50 nmol/L 25(OH)D]. Blood platelet concentrations [inflection at 39 nmol/L 25(OH)D] have previously been identified as being higher with low vitamin D status, perhaps as a result of higher inflammation, and the association of percentage of effector-memory CD8 T cells with 25(OH)D (inflection at 56 nmol/L) shows a similar pattern. In contrast, plasma eotaxin slopes increased with lower to higher vitamin D status (IP = 50 nmol/L), so this does not fit the same “higher inflammation at lower vitamin D status” pattern. The remaining 5 IPs in Figure 5 fell between 67 and 72 nmol/L 25(OH)D. Four of these involved activated CD4 T cells (percentages of CD4 PD1^+^ T cells, HLA-DR^+^ memory Tregs, and HLA-DR^+^ total Tregs) or memory CD4 T cells (percentage of memory Tregs), consistent with previous studies showing that vitamin D can dampen activation of inflammatory T cells as well as promote development of Treg cells. The fifth IP is for IP-10, a chemokine associated with the attraction of inflammatory cells, including T cells. Perhaps a useful message from these findings is that active inflammation (evidenced by high IL-6, MDC, platelets, and effector-memory CD8 T cells) is seen below ∼50 nmol/L 25(OH)D. Above this level, one can hypothesize benefits of vitamin D for maintaining appropriate adaptive immune activity, reaching a threshold at ∼70 nmol/L 25(OH)D at which Treg cell development and activity peak, representing appropriate regulation of inflammatory T-cell responses. Plasma IL-6 and MDC concentrations are also lower at 25(OH)D concentrations >70 nmol/L. Future experimental studies are needed to explore these associations.

### Lay summary

Vitamin D is essential for maintaining immune function. An adequate level of vitamin D status ensuring normal immune function is not known. We used serum 25-hydroxy vitamin D (25(OH)D) concentrations from healthy adults to identify linear and/or curvilinear associations between 25(OH)D and 79 markers of immune function with the goal of finding inflection points suggestive of a vitamin D status threshold for specific immune markers. Three inflection points were found at or below approximately 50 nmol/L, two of which relate to higher inflammatory immunity below this level. Five other inflection points were found at approximately 70 nmol/L, all of which suggest a threshold for dampening of inflammatory immunity peaking at this level, perhaps driven by regulatory T cells and dampening production of the T-cell chemokine IP-10. Four linear associations were also identified, three of which were consistent with the inflection point data suggesting dampening of immune activation at higher vitamin D status levels.

## Author contributions

The authors’ responsibilities were as follows – CBS, NR: conceived the study and designed scientific objectives; CBS, NR, RE-S: provided editorial and conceptual input to the final version of the manuscript; NR: performed the statistical analysis and literature review and composed the manuscript; and all authors: read and approved the final version of the manuscript.

## Data availability

Requests for data from the USDA ARS WHNRC Nutritional Phenotyping Study used in this analysis should be made via an email to the senior WHNRC author on the publication of interest. Requests will be reviewed quarterly by a committee consisting of the study investigators.

## Funding

This work was supported with funding from a USDA Agricultural Research Service (Project numbers 2032-51530-026-000-D, 2032-51530-025-00-D, 2032–53000–001-00-D, and 2032-51530-022-00-D) as well as the National Center for Advancing Translational Sciences (NCATS), and National Institutes of Health (NIH) through a grant, UL1 TR000002.

## Conflict of interest

The authors report no conflicts of interest.
